# Non-surgical Management of Colon Perforation Due to Recurrence of Colon Cancer With Extraluminal Feces in a Patient With Massive Peritoneal Adhesions

**DOI:** 10.7759/cureus.102997

**Published:** 2026-02-04

**Authors:** Tetsuya Toyoda, Keita Sakurai, Hideki Muramatsu, Yoshio Hashizume

**Affiliations:** 1 Choju Medical Institute, Fukushimura Hospital, Toyohashi, JPN; 2 Department of Radiology, National Hospital Organization Higashinagoya National Hospital, Nagoya, JPN; 3 Department of General Surgery, Toyohashi Municipal Hospital, Toyohashi, JPN; 4 Institute of Neuropathology, Fukushimura Hospital, Toyohashi, JPN

**Keywords:** colon cancer, colon perforation, fecal peritonitis, non-surgical management, peritoneal adhesion

## Abstract

Colon perforation with fecal peritonitis is usually fatal without prompt surgical intervention. We report the case of an 89-year-old woman with colon perforation and extraluminal feces who survived for 174 days without surgery in the presence of extensive postoperative peritoneal adhesions. The patient had previously undergone two abdominal surgeries for colorectal cancer, resulting in severe intra-abdominal adhesions. Despite radiological evidence of intraperitoneal free air and extraluminal feces, she was managed conservatively with antibiotics, morphine hydrochloride, supportive care, and close monitoring. This case suggests that, in carefully selected patients with marked peritoneal adhesions, non-operative management may warrant reconsideration as a potential therapeutic option for colon perforation before deeming that the condition is inoperable.

## Introduction

Colon perforation is associated with a high mortality rate, even with prompt surgical intervention, because it can rapidly progress to generalized peritonitis, sepsis, and disseminated intravascular coagulation (DIC) [1-3]. The choice of surgical strategy depends on multiple factors, including the etiology and location of the perforation, as well as the severity of peritonitis and the degree of intra-abdominal contamination [4].

In contrast, non-surgical management has been reported in selected cases of gastric or duodenal perforation with peritonitis [5-7]. In addition, a population-based study demonstrated a 90-day survival rate of 47.7% among patients aged 75 years or older who underwent non-operative management for perforated diverticular disease [8]. Conservative treatment has also been described as a feasible option for colon perforation caused by colonoscopy in carefully selected patients under close observation [9].

Colon perforation due to colorectal cancer remains a major clinical challenge [10]. When inflammation is severe, and primary anastomosis is considered unsafe, surgical management typically involves the creation of a temporary stoma, followed by reconstruction several months later after resolution of inflammation. In such cases, thorough intraoperative irrigation of leaked fecal material and purulent fluid, along with placement of drainage tubes, is essential to control intra-abdominal contamination.

To our knowledge, survival after colon perforation with fecal peritonitis and extraluminal feces secondary to colon cancer, managed entirely without surgery, has not been previously documented. Here, we report such a unique case.

## Case presentation

An 89-year-old woman with Alzheimer’s disease residing in a nursing home presented to the outpatient clinic of Fukushimura Hospital with vomiting, abdominal pain, fever (39.2 °C) and impaired consciousness (Japan Coma Scale score: 100). Physical examination revealed signs of peritoneal irritation and a bulge in the left lower abdomen. Her medical history included open surgery for transverse colon cancer in 2017 (at 82 years of age) and surgery for rectal cancer with temporary colostomy in 2012 (at 77 years of age), both performed at Toyohashi Municipal Hospital.

**Figure 1:**
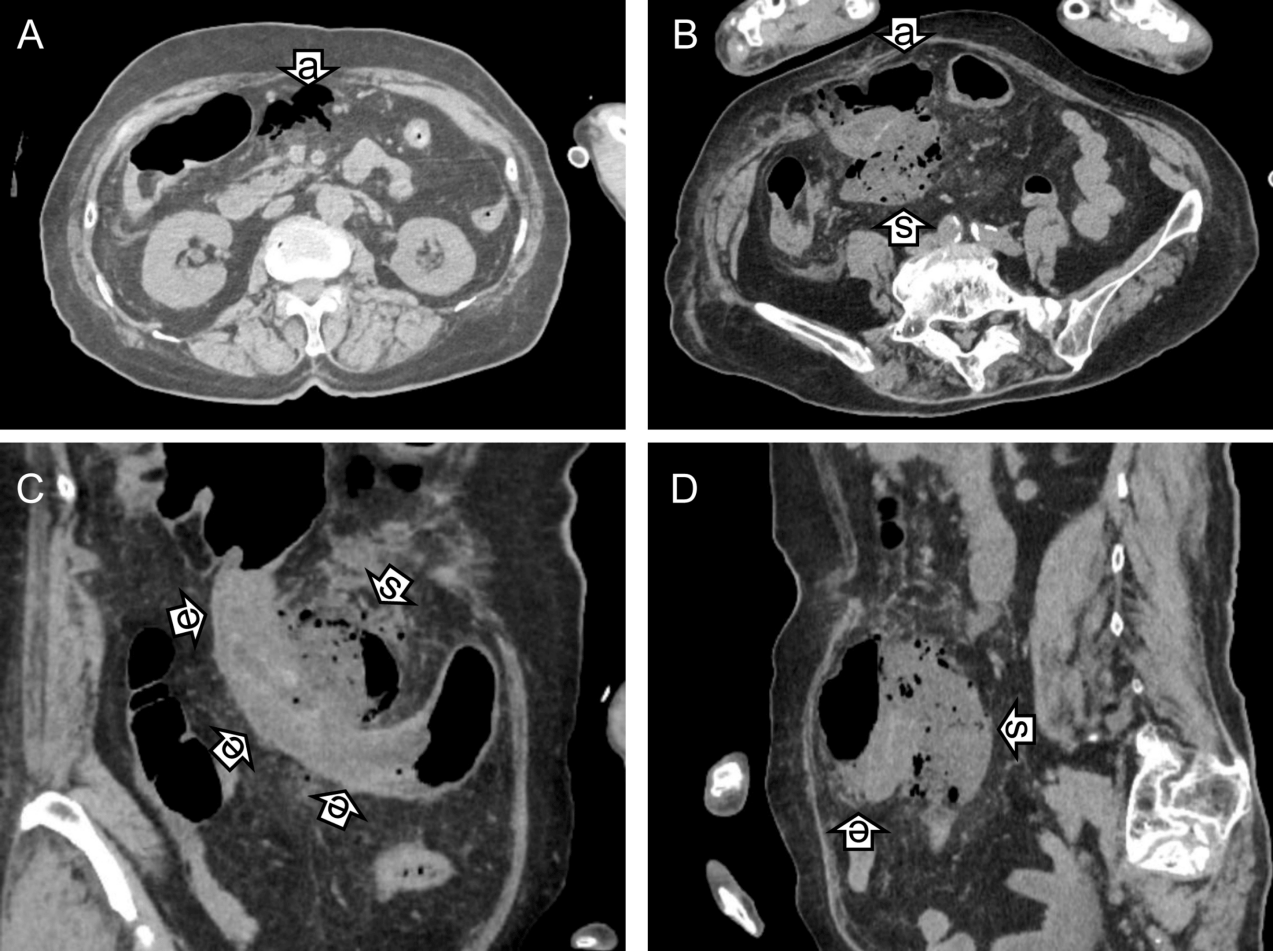
Computed tomography findings on day 2. On day 2, free air (arrows “a”, panels A and B), extraluminal feces (arrows “s”, panels B–D), and edematous transverse colon (arrows “e”, panels C and D) were evident on axial and coronal CT images.

Computed tomography (CT) demonstrated free intraperitoneal air, extraluminal feces, herniation of the omentum into a ventral hernia cavity, and colon perforation with fecal spillage resulting in peritonitis (Figure 1A-1D). The clinical course is summarized in Table 1. Marked leukocytosis and C-reactive protein (CRP) elevation were observed, followed by gradual improvement.

**Table 1:** Clinical course On day 2, WBC and CRP values were the highest. Hematochezia developed on day 9 and persisted until day 30, but the Hb level remained above 10 g/dL until day 16. -, Laboratory test not performed on that day. *, Therapy (T/P, morphine hydrochloride, oxygen therapy, or TPN) was administered continuously during the intervening days. T/P was administered from day 1 to 27. Morphine hydrochloride was administered from day 1 to 7. TPN was administered from day 17 to 174. Decompression continued from day 1 to 5. Oxygen was administered from day 1 to 10, and from day 34 to 174. WBC, white blood cells; RBC, red blood cells; Hb, hemoglobin (values < 7 g/dL are shown in italics); Hct, hematocrit; Pl, platelet; CRP, C-reactive protein (values > 10 mg/dL are shown in bold); EBL, erythroblast cells; T/P, tazobactam piperacillin hydrate; TPN, total parenteral nutrition.

Variable	Day 1	Day 2	Day 7	Day 8	Day 9	Day 10	Day 14	Day 16	Day 17	Day 21	Day 27	Day 30	Day 34	Day 44	Day 50	Day 62	Day 66	Day 78	Day 106	Day 115	Day 118	Day 128	Day 146	Day 174
WBC (/µL)	6080	17320	10180	-	15530	-	12710	14460	-	9440	6410	6250	7290	10250	9390	25030	7020	8540	8560	3480	4590	4560	5810	-
RBC (10^4^/µL)	414	370	352	-	336	-	344	331	-	297	282	262	252	231	212	195	192	212	232	196	195	173	143	-
Hb (g/dL)	12.6	11.2	10.5	-	10.1	-	10.3	10	-	8.9	8.5	8.1	7.6	6.9	6.2	5.9	5.8	6.5	7.2	6.2	6.1	5.5	4.9	-
Hct (%)	38.2	35.4	33.6	-	31.7	-	33.3	33.1	-	30.6	29.7	27.9	25.2	23.2	20.3	18.6	19.3	22.1	26.8	20.6	20.1	17.9	15.4	-
Pl (10^4^/µL)	24.3	20.8	19.1	-	25.6	-	28.7	28.5	-	19.5	25.1	21.4	16.9	15.9	24.2	23.7	25.2	30.6	26	15.6	16.1	11.8	19.9	-
CRP (mg/dL)	1.57	26.22	18	-	-	-	4.59	4.73	-	6.38	3.76	4.75	7.43	17.53	6.09	8.03	3.02	2.41	6.13	2.55	1.83	-	1.96	-
Myelocyte (%)	0	4	4	-	1	-	0	1	-	0	0	0	0	0	0	0	0	0	0	0	0	0	0	-
Metamyelocyte (%)	0	3	2	-	0	-	0	0	-	0	0	0	0	0	0	0	0	0	0	0	0	0	0	-
EBL/100WBC	0	0	0	-	0	-	0	0	-	0	0	0	0	0	0	0	0	0	0	0	0	0	0	-
T/P	*	*	*	*	*	*	*	*	*	*	*	-	-	-	-	-	-	-	-	-	-	-	-	-
Morphine	*	*	*	-	-	-	-	-	-	-	-	-	-	-	-	-	-	-	-	-	-	-	-	-
TPN	-	-	-	-	-	-	-	-	*	*	*	*	*	*	*	*	*	*	*	*	*	*	*	*
Decompression	*	*	*	*	-	-	-	-	-	-	-	-	-	-	-	-	-	-	-	-	-	-	-	-
Oxygen therapy	*	*	*	*	*	*	-	-	-	-	-	-	*	*	*	*	*	*	*	*	*	*	*	*

Considering the patient’s advanced age, impaired level of consciousness, presumed severe intra-abdominal adhesions that appeared to confine the extraluminal feces and free air, the anticipated technical difficulty of surgery, and the absence of an apparent abscess, surgical intervention was not pursued. After detailed discussions with the patient’s family, a palliative approach focused on relief of pain and distress was selected because death due to disseminated intravascular coagulation (DIC) was considered highly likely at presentation.

On admission, intravenous tazobactam/piperacillin (2.25 g three times daily) and morphine hydrochloride (10 mg three times daily) were initiated. Morphine was reduced to twice daily on day 3 and discontinued on day 7. Gastric decompression with a nasogastric tube was maintained until day 7, and oxygen therapy continued until day 10.

Hematochezia developed on day 9 and persisted until day 30 (Table 1), prompting administration of carbazochrome sodium sulfonate (100 mg/day) and tranexamic acid (250 mg/day). Blood transfusion was not performed after discussion with the family because the patient was elderly, bedridden, and unable to tolerate oral intake. Oxygen therapy was reinitiated on day 34 when the hemoglobin level declined to 7.6 g/dL. Although overt hematochezia subsequently resolved, anemia progressed, with hemoglobin decreasing to 4.9 g/dL by day 146.

**Figure 2:**
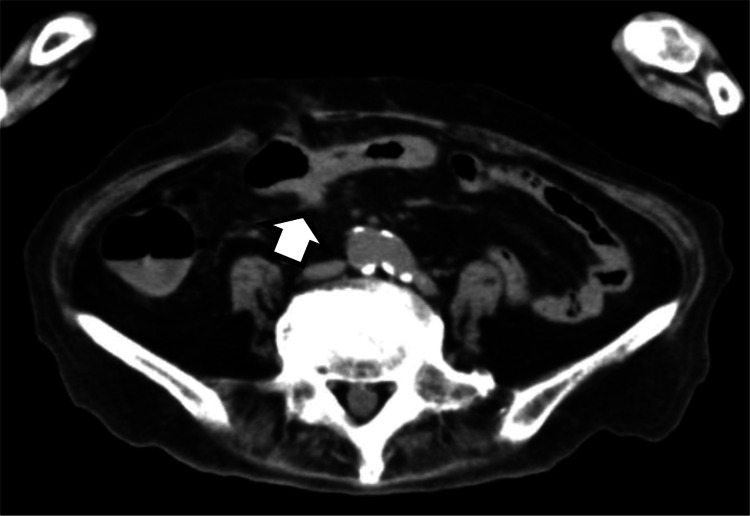
Computed tomography findings on day 106. By day 106, edema of the transverse colon and other abnormalities, including extraluminal feces, which were demonstrated in Figure 1B had resolved, and a small residual soft-tissue density persisted (arrow). See Figure 1B (arrow “s”). This CT was taken at the almost identical slice position as Figure 1B.

Total parenteral nutrition (TPN) was initiated on day 17 after the patient survived beyond the initial prognosis. Follow-up CT on day 107 showed complete resolution of the extraluminal feces (Figure 2).

**Figure 3:**
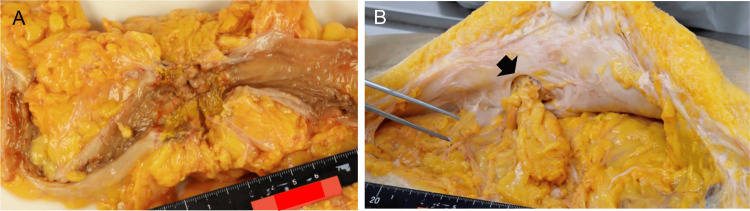
Autopsy findings. Recurrent cancer with luminal stenosis was observed at the prior transverse-colon surgical site (panel A). An internal hernia in the left flank peritoneum (arrow), distant from the surgical site, exhibited omental impaction without involvement of the large intestine (panel B).

The patient died of heart failure on day 174, and a general autopsy was performed. Pathological examination revealed severe postoperative adhesions, recurrence of transverse colon cancer with luminal stenosis at the previous surgical site (Figure 3A), a moderately differentiated adenocarcinoma invading the serosa, and a small metastatic lesion (approximately 1 cm in diameter) in the left hepatic lobe. No evidence of cancer recurrence, luminal stenosis, or liver metastasis had been detected on CT during the clinical course. An internal hernia with omental impaction was identified in the left flank peritoneum, distant from the prior surgical site (Figure 3B). No evidence of ongoing peritonitis or intestinal perforation was observed, suggesting that the clinically detected perforation had likely originated at the site of tumor recurrence. The cause of death was confirmed as heart failure.

## Discussion

Peritonitis resulting from colon perforation is generally life-threatening and associated with a high mortality rate [1,3]. A PubMed search using the terms “colon perforation,” “fecal peritonitis,” and “non-operative” or “non-surgical management” identified no previous reports of survival following colon perforation with fecal spillage managed without surgery, except in cases related to diverticulitis.

Patients with advanced dementia are often poor candidates for surgical treatment under general anesthesia. This exceptionally rare case suggests that extensive postoperative adhesions involving the omentum, small intestine, and colon may compartmentalize fecal contamination and limit the spread of infection. The incidence of peritoneal adhesions after colorectal cancer surgery in Japan has been reported to be approximately 20-30% following open surgery [11]. Elderly patients, in particular, have a markedly increased risk of mortality, even when perforation occurs as a complication of colonoscopy [12].

In the present case, broad-spectrum antibiotic therapy and effective pain control with morphine, which reduces intestinal peristalsis, may have contributed to survival. Earlier initiation of TPN and prompt blood transfusion might have further improved the clinical course; however, palliative care was initially selected based on the patient’s extremely poor prognosis at presentation. Although such cases are exceedingly rare, this report suggests that non-surgical management may represent a possible option for patients who are unsuitable surgical candidates, provided that extensive abdominal adhesions effectively limit the spread of contamination.

## Conclusions

Colon perforation with fecal peritonitis is usually fatal without prompt surgical intervention. This report describes a rare instance of successful non-surgical management of colon perforation with fecal peritonitis secondary to recurrent colon cancer. In this case, extensive postoperative abdominal adhesions may have compartmentalized fecal contamination and limited the dissemination of infection. This case highlights the potential role of conservative therapy in carefully selected patients, particularly elderly individuals with significant comorbidities and complex surgical histories. Accumulation of similar cases through prospective documentation is required to further evaluate this rare treatment strategy. Finally, the importance of multidisciplinary consultation and family involvement in treatment decision-making is emphasized.
